# Methane-oxidizing bacterial community dynamics in sub-alpine forest soil

**DOI:** 10.1128/spectrum.00834-24

**Published:** 2024-09-17

**Authors:** Delaney G. Beals, J. Jackson Munn, Aaron W. Puri

**Affiliations:** 1 Department of Chemistry and the Henry Eyring Center for Cell and Genome Science, University of Utah, Salt Lake City, Utah, USA; Instituto de Ecología, A.C. (INECOL), Pátzcuaro, Michoacán, Mexico

**Keywords:** methane flux, methanotrophs, methylotrophs, 16S rRNA, forest soil

## Abstract

Microbial activities in sub-alpine forest soil influence global cycling of the potent greenhouse gas methane. Understanding the dynamics of methane-oxidizing bacterial communities, particularly the roles of potentially active versus total microbial populations, is necessary for reducing uncertainty in global methane budget estimates. However, our understanding of the factors influencing methane cycling in forest soils is limited by our lack of knowledge about the biology of the microbes involved and how these communities are shaped by their environment. Here, we compared the composition and potential activity of microbial communities using 16S rRNA gene amplicon sequencing of total genomic DNA (gDNA) and potentially active complementary DNA (cDNA) from shallow soil in Red Butte Canyon (Salt Lake City, Utah, USA). We compared riparian and upland soils at two time points in the growing season and found distinct differences in both the community composition of the gDNA and cDNA libraries and the potential drivers of these community structures. Aerobic methane-oxidizing bacteria (methanotrophs) were detected in all samples, with cDNA libraries containing a higher average relative abundance and diversity of methanotrophs compared to gDNA libraries. Methane flux at the sample sites did not significantly correlate to the relative abundance (gDNA) or potential activity (cDNA) of methanotrophs. In the cDNA libraries, there were significant positive correlations between the abundance of *Methylococcaceae* family methanotrophs and several non-methanotrophic methylotrophs previously found to be associated with methane-oxidizing bacterial communities. These findings suggest a complex relationship between methane-cycling bacterial communities and methane flux and highlight the need for further *in situ* studies to understand the environmental and ecological influences of these microbial consortia.

**IMPORTANCE:**

Methane-oxidizing bacteria are found in diverse soil and sediment environments and play an important role in mitigating flux of this potent greenhouse gas into the atmosphere. However, it is unclear how these bacteria and their associated communities are structured in the environment and how their activity ultimately influences methane flux. In this work, we examine the composition and structure of methane-oxidizing bacterial communities in sub-alpine forest soil and find soil- and time-specific differences between the stable and potentially active populations. We also find that the potentially active populations of certain methanotrophs and non-methanotrophs are positively correlated. This work provides a step toward refining our understanding of microbially mediated biogeochemical cycles.

## INTRODUCTION

Biogeochemical processes largely determine whether soils contribute to or mitigate the impact of greenhouse gases on the atmosphere (1, 2). In the United States, sub-alpine forests are widely distributed and play an important role in carbon exchange in areas with high forest coverage (3, 4). Organic carbon turnover in soil is affected by temperature and precipitation as a result of changes in microbial activity (5–7). Soil organic matter is cycled, in part, by methanogenic archaea, which anaerobically produce methane under water saturated, reducing conditions as a product of metabolism (8–10). Methane released from soil contributes to total global methane emissions (9–11) but can be significantly offset by the biofiltering activities of aerobic methane-oxidizing bacteria (12, 13). These bacteria, known as methanotrophs, use methane as a carbon and energy source and are found in environments with access to both oxygen and methane, such as the top centimeters of soil and sediment (12, 14, 15). Together, the abundance and composition of methane cycling microbial communities shape methane flux direction and magnitude in soil, which affects the degree to which biogenic sources affect total methane content in the atmosphere (16).

The majority of methanotrophs belong to either the Alphaproteobacteria or Gammaproteobacteria classes within Pseudomonadota (synonym Proteobacteria), though recent efforts have identified methanotrophic microorganisms that fall outside these taxonomic groups (17, 18). Despite their importance in mitigating methane flux, methanotrophic bacteria are typically represented in low relative abundances in soil (14). Additional research has highlighted the potential role of non-methanotrophic bacteria in the methane-mitigating function of soil microbiomes. In particular, bacteria that can use reduced, one-carbon compounds besides methane, here referred to as non-methanotrophic methylotrophs (NMMs), have been reported to feed off the inhibitory byproducts of methanotrophic metabolism (e.g., methanol) (19–24). This web is further extended by the presence of a broader group of non-methylotrophic heterotrophs (NMHs) that can associate with methane-cycling community members through cross-feeding of methane-derived carbon and specific nutrients (20–29).

Metabolic partnerships between different functional guilds within methane-oxidizing bacterial communities have been found through lab-based enrichments and stable isotope probing (19, 26, 30). For example, methane-fed microcosms of Lake Washington sediment revealed that communities were dominated by methanotrophic bacteria from the family *Methylococcaceae*, NMMs from the family *Methylophilaceae*, and other specific NMHs (20, 22–26). Despite the diversity of microorganisms within the sediment inoculum, including methanotrophs, NMMs, and NMHs from additional taxa, the methane-oxidizing activity of methanotrophs resulted in a reproducible community structure. An ongoing question regarding these metabolic partnerships is whether they develop only as a result of co-occurrence of the partners in question, or whether more specific processes, beyond metabolic crossfeeding, drive these relationships.

While specific metabolic partnerships between different functional guilds have been reported in methane-fed, laboratory-based studies, there have been relatively few reports on the potential of these relationships *in situ*. Sequencing of 16S rRNA gene transcripts can serve as a measure of protein synthesis potential in members of a microbial community (31). Paired with 16S rRNA gene sequencing data derived from genomic DNA (gDNA), the relative abundance of transcripts can be used to identify community members with the potential grow and acclimate at a given timepoint in a range of ecosystems, including within methane-oxidizing bacterial communities in soil. To this aim, we performed a pilot study employing 16S rRNA gene amplicon sequencing based on gDNA and complementary DNA (cDNA, synthesized from total RNA) to investigate the methane-cycling microbial community in the upper soil layer at a sampling site within the Red Butte Canyon Research Natural Area in Salt Lake City, Utah, which is subject to ongoing climate, aquatic, and soil monitoring (32, 33).

## MATERIALS AND METHODS

### Study site and soil sampling

Soil sampling and methane flux measurements were conducted in the growing season (May through October, excluding the month of September) in the Knowlton Fork area of Red Butte Canyon (40°48′36″N 111°45′56″W) in Salt Lake City, Utah, at an elevation of approximately 1,990 m above sea level. Starting in May 2021, we collected a 500 cm^3^ (10 × 10 × 5 cm) soil sample from each of the two soil types within 0.5 m of the accompanying methane flux measurement chambers. Soil was collected after the completion of all methane chamber sampling. Soil samples were placed in a cooler with ice packs and transported back to the University of Utah within 2 h. Bulk soil was aliquoted and stored at −80°C for up to a week before total RNA extraction and up to 3 months before genomic DNA extraction.

### Soil environmental measurements

Soil temperature was recorded at a depth of 5 cm with a probe thermometer at each gas sampling time point in soil adjacent to each methane flux chamber. Environmental data from the Knowlton Fork remote research station were downloaded from the publicly available database *Wasatch Environmental Observatory Red Butte Network: Raw Data at Knowlton Fork Climate (RB_KF_C)* for the year 2021 (34). Data values for the parameters air temperature, soil temperature, relative plant height, and volumetric water content (VWC) were reported here as averages from four measurements from the 1 h active methane sampling time period. Gravimetric water content (GWC) was determined by weighing soil aliquots and then drying in a 60°C oven until their masses no longer changed. The mass of water lost was then divided by the mass of dry soil.

### Soil methane flux measurements

To measure methane flux from Red Butte Creek soil, we used static, non-steady state enclosures (35). Three 0.032 m^2^ circular PVC collars were placed within 1 m from each other at both the riparian and upland zones. PVC collars were inserted 5–10 cm into the soil and left to equilibrate for 1 h before securing a PVC cap. Every 15 min for 1 h, 15 mL of chamber air was extracted through the PVC cap septum using a 30-mL syringe fitted with a one-way stopcock and 23-gauge needle. All 15 mL of sample was injected into pre-evacuated 12 mL Exetainer vials (Labco) to minimize sample loss across the vial septa. Atmospheric air and pre-filled vials of 100% CH_4_ samples were collected using a similar method throughout the hour-long sampling process for comparison to chamber concentrations. Vials containing gas samples were stored in a cooler with ice packs during transport to the University of Utah and kept at 4°C until analysis within 3 days.

### Methane quantification

Gas samples were analyzed on an Agilent 6890N gas chromatograph by flame ionization detection. A 30 m × 0.32 mm column with a thickness of 0.25 µm was used for sample separation with argon carrier gas and a split ratio of 20:1. The oven temperature was set at 35°C, the inlet at 200°C, and the detector at 250°C. Methane standards (Supelco), balanced with N_2_, were used to generate calibration curves to convert the resulting peak area to ppm. Methane flux rates from each chamber were calculated by first converting ppm to mass using the Ideal Gas Law and then plotting the mass of methane detected in the first three sampling points versus time (35). Linear regressions were performed to determine the linearity of flux, and the slope of the line was divided by the cross-sectional area of the collars to obtain final methane flux rates in μg CH_4_-C h^−1^ m^−2^.

### Nucleic acid extraction and 16S rRNA gene sequencing

Three replicate soil aliquots each were used to extract DNA and RNA from the same bulk soil sample in June and October. DNA was extracted from ~500 mg of soil per sample using the FastDNA Spin Kit for Soil (MP Biomedicals). PCR amplification was performed using the Earth Microbiome Project protocol (36) with 16S rRNA gene primers Parada 515F/Apprill 806R with Illumina adapters added to the 5′ end of each primer. Gel electrophoresis was used to confirm the correct size of resulting amplicons (approximately 390 base pairs including the sequencing adapters). PCR products were purified using the GeneJET Gel Extraction Kit (Thermo Fisher Scientific). Samples were normalized to a concentration of 20 ng/µL before 2 × 250 bp Illumina NovaSeq sequencing through the Amplicon EZ service (Genewiz, Azenta Life Sciences), with a throughput of approximately 50,000 reads per sample.

Total RNA was extracted from ~500 mg of soil per sample using the FastRNA Pro Soil-Direct Kit (MP Biomedicals) and subsequently treated with Ambion DNase I (Invitrogen). To check for DNA contamination, PCR amplification of purified RNA samples was performed using iProof High-Fidelity PCR Kit (Bio-Rad) and primers 27F/1492R, which amplify the 16S rRNA gene of bacteria (37). Reactions were set up following the manufacturer’s protocol and ran at 98°C for 30 s, denatured at 98°C for 10 s, annealed at 61°C for 20 s, and extended at 72°C for 60 s for a total of 30 cycles, with a final extension at 72°C for 5 min. PCR products were analyzed by gel electrophoresis, and the presence or absence of a band in RNA samples was compared to reactions containing a positive control template (dilute genomic DNA from the methanotroph *Methylomonas* sp. LW13) and a negative control template (RNase-free water). RNA samples resulting in a band on the gel electrophoresis were re-treated with DNase I and checked again for DNA contamination. RNA concentrations were quantified using the Qubit RNA High Sensitivity assay kit (Invitrogen). Complementary DNA (cDNA) was synthesized from RNA samples of equal concentration using iScript Reverse Transcription Supermix (Bio-Rad). PCR amplification and sequencing of the 16S rRNA gene region from cDNA samples were conducted using the same protocol performed for gDNA templates.

### Data processing

Data processing was performed using R Statistical Software (v4.3.0) (38). Amplicon sequence processing was performed using the DADA2 platform for R (dada2 package, v1.28.0) (39). Sequences were trimmed for quality and to remove primers before the generation of a library-specific error model. This error model was used for dereplication, sample inference, and merging paired reads. *De novo* chimera removal was performed before assigning taxonomy to the resulting amplicon sequence variants (ASVs) using the Ribosomal Database Project (RDP) training set (v18) formatted for DADA2 (39, 40). Rarefaction plots were generated to assess and confirm that samples were sequenced with sufficient coverage to reach convergence (Fig. S1). To account for different sequencing depths, multiple normalization techniques were applied separately depending on the desired analysis. The relative abundances of ASVs were used for visualization of microbial communities. Rarefaction to the lowest sampling depth with replacement was performed prior to generating Bray-Curtis distance matrices (41).

### Statistical analysis and data visualization

Based on 16S rRNA gene sequences, the following genera detected in our samples were considered methanotrophic bacteria: *Methylobacter*, *Methylomonas*, *Methylosinus* (42)*, Methyloferula* (43)*,* and *Methylomicrobium* (44). Genera found in our study that included NMMs relevant to methane cycling were as follows*: Methylophilus* (45), *Methylobacterium* (46), *Methyloversatilis* (47), *Methylotenera*, *Methylibium* (48)*, Methylorubrum* (49), *Methyloceanibacter*, *Methyloligella*, *Methylopila* (50). NMHs included were *Acidovorax* (26), *Comamonas* (51), and *Flavobacterium* (25).

Statistical analysis and data visualization were performed using R Statistical Software (v4.3.0) (38). Pearson correlation tests were used to examine the strength of the relationship between two different continuous variables, including the relative abundances of varying taxonomic classifications and environmental data. We calculated Hill numbers (effective numbers of diversity) based on ASV count tables and tested for differences in month and soil type using the hill_div function and div_test function in the hilldiv package (v1.5.1) for R (52). Bray-Curtis dissimilarity distance matrices were generated using the avgdist function in the vegan package (v2.6.4) for R (53). NMDS ordinations were constructed using the metaMDS function (vegan), and the statistical difference between gDNA and cDNA distances was computed using PERMANOVA in the adonis2 function (vegan). Differential expression analysis between nucleic acid template libraries was conducted using DESeq2 (v1.40.2) (54).

## RESULTS AND DISCUSSION

### Methane emission and environmental parameters in Red Butte Canyon

**Fig 1:**
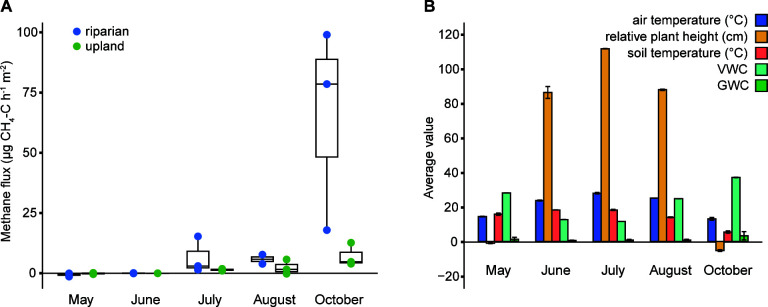
(**A**) Methane flux at riparian and upland soil sample sites (*n* = 3 for each soil type). No measurements between samples are significantly different. (**B**) Environmental data were measured by a remote research station at the time of methane sampling (*n* = 4). VWC is volumetric water content; GWC is gravimetric water content. Bars show the mean ± one standard deviation.

The Knowlton Fork sampling site in Red Butte Canyon is located at the confluence of two creeks and was selected due to its intermittent water saturation and high plant turnover (Fig. S2). We sampled the saturated creek bank (riparian) and higher up a hillside several meters away (upland), allowing us to compare the differences in methane flux and microbial communities while minimizing additional variables that arise when sampling across a larger overall landscape (55). From May to October, methane flux measurements across riparian and upland varied from −1.40 to 99.0 µg CH_4_–C m^−2^ h^−1^, but these variations were not statistically significant (Fig. 1A). We observed that starting in July, median methane flux was higher in riparian soil than upland soil, but the significance of this difference was impacted by high variability between replicate chambers. Intra-chamber soil temperature and methane flux values did not correlate, while the median methane flux from the two soil types across 5 months of sampling had a significant negative correlation (*R* = −0.96, *P* < 0.01) with the 5 cm soil temperature recorded by a continuously monitored remote research station several meters away in upland soil (Fig. 1B; Fig. S3). However, this result may be driven by a single lower temperature datapoint, and further data are needed before this correlation is convincing.

We did not find significant correlations between methane flux and volumetric water content, air temperature, plant cover, or gravimetric water content which are factors previously reported to influence soil methane flux in some environments (16, 56). The lack of significant correlation between methane flux and these soil factors in Red Butte Creek could indicate that other variables, such as organic matter content (57, 58) or microbial community composition (59), are drivers of methane flux variability. Furthermore, the relatively small sample sizes used in this pilot study may not be sufficient to statistically determine the strength of correlations between methane flux and abiotic soil factors.

### Overall microbial community composition

We examined soil microbial communities from the riparian and upland soil sites collected in June and October due to differences in measured environmental parameters at these timepoints (Fig. 1). Three replicates each of DNA and RNA extracts were used to generate 16S rRNA gene amplicon sequencing libraries to compare the compositions of stable and potentially active microbial populations within the soil. Optimal RNA preservation methods, such as shock freezing during sampling or adding an RNA stabilizing agent, were not employed in this study. Although rRNA is generally more stable than mRNA, the lack of these preservation methods may have affected the stability of the rRNA and potentially impacted the rRNA (cDNA) transcript analysis results. A total of 31,249 ASVs were identified across all 24 samples, with individual samples containing up to 6,820 observed ASVs at the most (October upland cDNA) and 1,626 observed ASVs at the fewest (June riparian cDNA).

**Fig 2:**
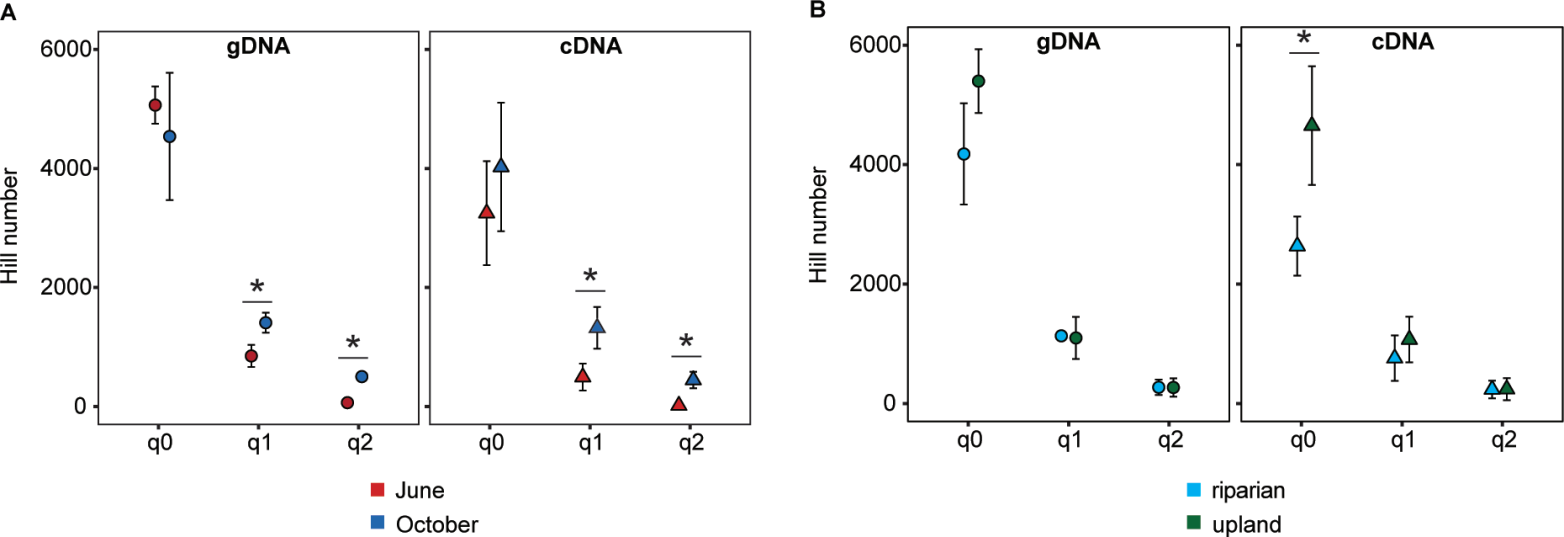
Hill numbers (ASV diversity) at increasing orders of diversity between nucleic acid libraries of microbial communities from (**A**) months June and October or (**B**) riparian and upland soil types. *, statistically significant difference between points (*P* < 0.05).

When analyzing gDNA and cDNA sequence libraries separately, we found that in both the stable and potentially active communities, June and October soils contained similar mean numbers of ASVs (*α* = 0.05) at the q0 order of diversity, an alpha diversity metric that represents raw richness by weighting rare ASVs the same as abundant ASVs (Fig. 2A). At higher diversity orders q1 (ASVs weighted by their frequency but not abundance) and q2 (abundant ASVs overweighted), October soils were more diverse than June in both gDNA and cDNA groups (*P* < 0.05). This result suggests that while the overall species richness (q0) remains steady between different months, the evenness and dominance patterns (reflected by q1 and q2) of stable and potentially active communities are significantly affected by environmental changes associated with the month.

In a similar analysis, we compared the diversity of ASVs between riparian and upland soil at multiple orders of diversity (Fig. 2B). In gDNA libraries, there was no significant difference between the alpha diversity of riparian and upland soils at q0, q1, or q2. This result suggests that the two soil types had similar community composition and structure even when including both common and rare ASVs. In cDNA libraries, upland soil had a significantly higher (*P* < 0.05) species richness (q0) than riparian soil, but this difference was not observed at q1 and q2 orders of diversity. The higher species richness in upland soil cDNA libraries suggests that a greater variety of potentially active microbial species are present in upland soil compared to riparian soil. The lack of differences at q1 and q2 in cDNA libraries indicates that while more species are potentially active in upland soil than riparian soil, their relative abundances and dominance patterns are similar in both soil types and that species uniquely represented in upland cDNA libraries are not abundant enough to significantly impact community evenness and dominance.

**Fig 3:**
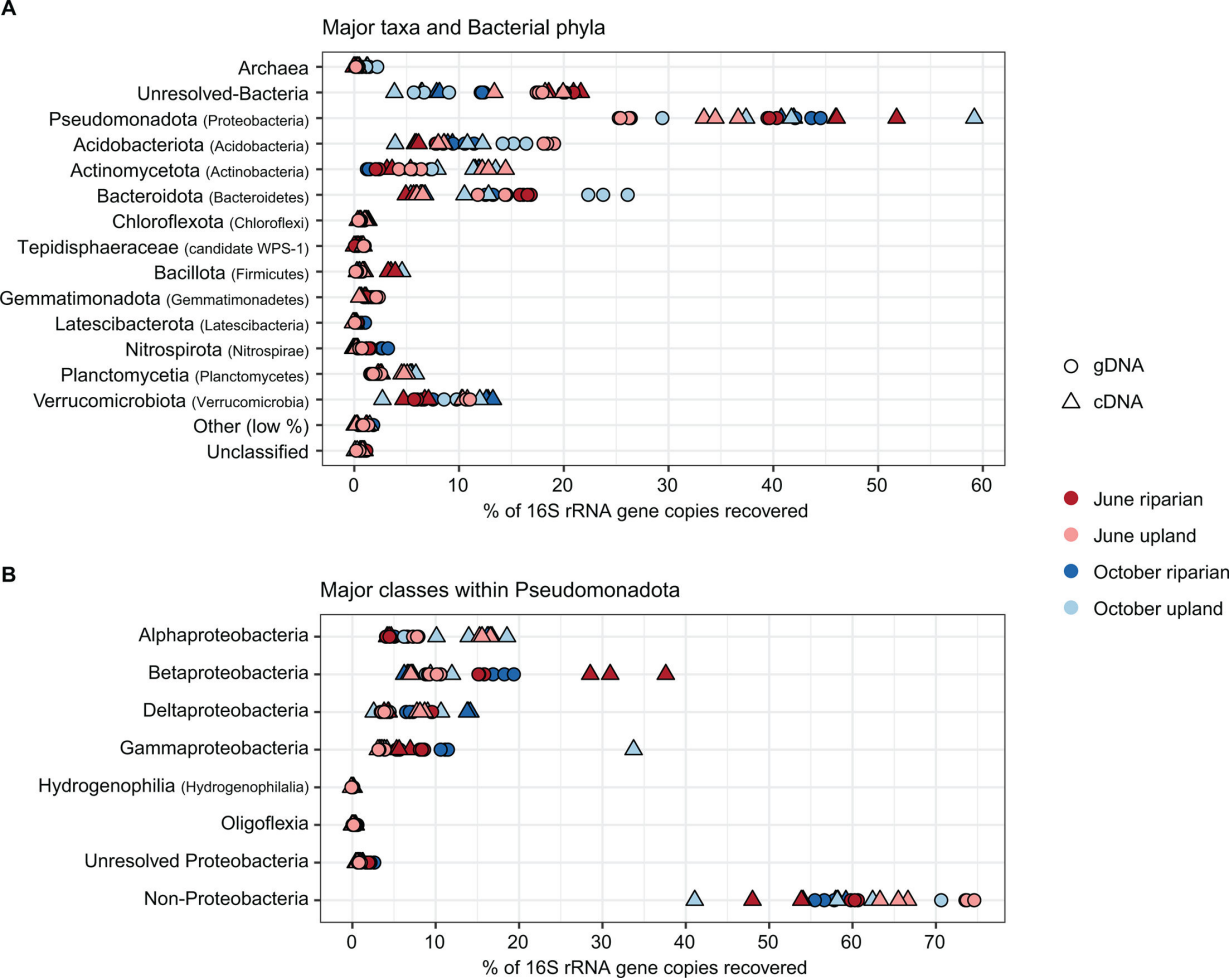
(**A**) Relative abundance of bacterial phyla from gDNA- and cDNA-derived sequence libraries of soil. (**B**) Relative abundance of taxonomic classes within Pseudomonadota. Synonyms of taxonomic names are included in parentheses.

The composition of the microbial communities based on relative abundance varied between soil types and months but followed a similar distribution at the phylum level (Fig. 3A). In all sequence libraries, Pseudomonadota had the highest relative abundance, ranging from 25.3% to 44.5% for gDNA samples and 33.5% to 59% for cDNA samples. Within Pseudomonadota, the most abundant class was Betaproteobacteria, ranging from 8.8% to 19.2% in gDNA samples, and 6.26% to 37.6% in cDNA samples (Fig. 3B). One nucleic acid extraction replicate from the October upland cDNA group had disproportionately higher counts of ASVs within the Gammaproteobacteria orders Enterobacterales and Methylococcales, the latter of which includes the methanotrophic genera *Methylobacter* and *Methylomonas*. However, the number of ASVs, proportions of other taxa, and number of reads for this sequence library were commensurate with other samples, so it was included in this study.

The relative abundance of Archaea for any sample was less than 3%, with Nitrososphaerota (synonym Thaumarchaeota) identified as the most abundant archaeal phylum in all sequence libraries. Within the Nitrososphaerota phylum, 30 of 36 ASVs were assigned to the *Nitrososphaera* genus, which are aerobic ammonia-oxidizing archaea commonly found in soil (Fig. S4) (60). No methanogenic (methane-producing) archaeal taxa were detected in any sample at the 5 cm soil depth, which was confirmed using four common 16S rRNA gene taxonomic databases (RDP v18, Silva v138.1, GTDB v202, and RefSeq/RDP v16) (40, 61–65). This low abundance aligns with previous findings reporting that archaea generally constituted 0%–2% relative abundance of shallow sediment microbial communities and did not contain methanogens (66). Additionally, shallow aerated soils are also commonly dominated by non-methanogenic archaea (67).

**Fig 4:**
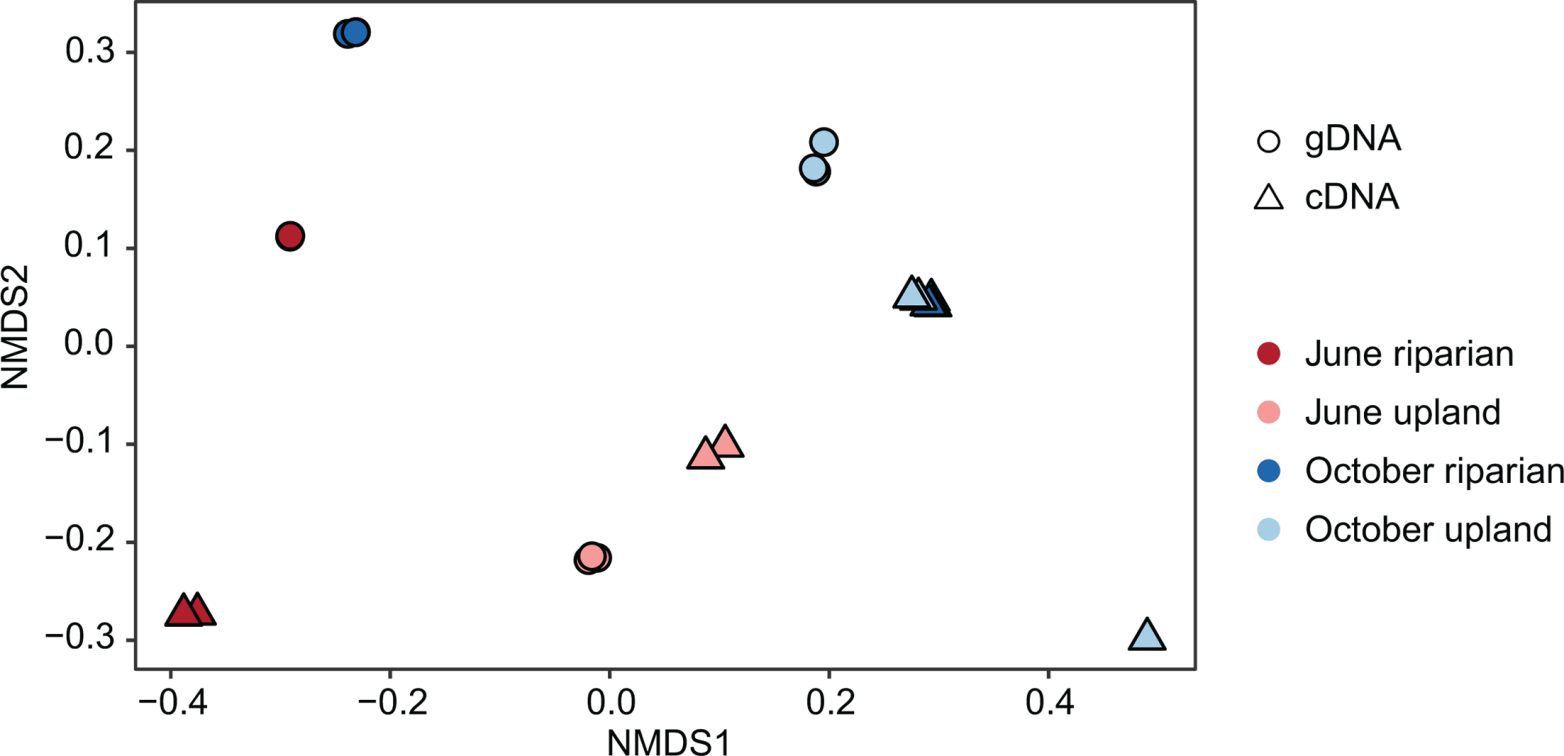
NMDS ordination of bacterial community composition in riparian and upland soil from June and October. Points represent microbial community dissimilarity calculated from Bray-Curtis distances of sequence libraries generated from triplicate DNA and RNA extractions from soil.

To examine the spatial and temporal differences between microbial communities more broadly, we used non-metric multidimensional scaling (NMDS) ordination to examine the relative relatedness of each soil community (Fig. 4). Ordination plots showed that communities from replicate libraries clustered closely to one another (PERMANOVA; *P* < 0.01), suggesting that the three soil aliquots used for extracting either gDNA or RNA contained relatively similar microbial representatives. Microbial communities from gDNA and cDNA libraries did not cluster together (PERMANOVA; *α* = 0.05). This distinction suggests that the permanent (gDNA) and potentially active (cDNA) microbial communities differ and that detection of 16S rRNA genes in gDNA is not necessarily coupled to the detection of gene transcripts in the cDNA libraries. To this end, for most individuals within the methane-oxidizing community group (Table S1), there was no significant correlation (Pearson correlation, *α* = 0.05) between their abundances in gDNA and cDNA libraries (Fig. S5). These findings indicate that the factors driving the stable and potentially metabolically active communities may be separate and highlight the importance of directly assessing the potentially active portion of a microbial community.

The NMDS ordination further allowed us to determine the drivers of differential abundances found within gDNA and cDNA library groups. The gDNA libraries could be distinguished between the two soil types, rather than the time of year, whereas among cDNA samples, sequence libraries were divided between the 2 months (Fig. 4). Statistical analysis of dissimilarity distance matrices confirmed significant differences (PERMANOVA, *P* < 0.005) between gDNA communities from riparian and upland soils, and between cDNA communities from June and October. These distinctions suggest that the stable microbial populations in each soil sample likely develop based on their landscape position, which is either riparian or upland in this study. At small spatial scales, the distribution of soil microbes has previously been found to be shaped by relatively fixed terrain attributes such as slope position (68, 69), soil pH (70), or aboveground vegetation (71, 72). Alternatively, the potentially active microbial populations measured by cDNA sequencing may be more influenced by month-to-month changes in surface-level conditions, such as precipitation or air temperature.

### Methane-oxidizing bacterial community compositions

**Fig 5:**
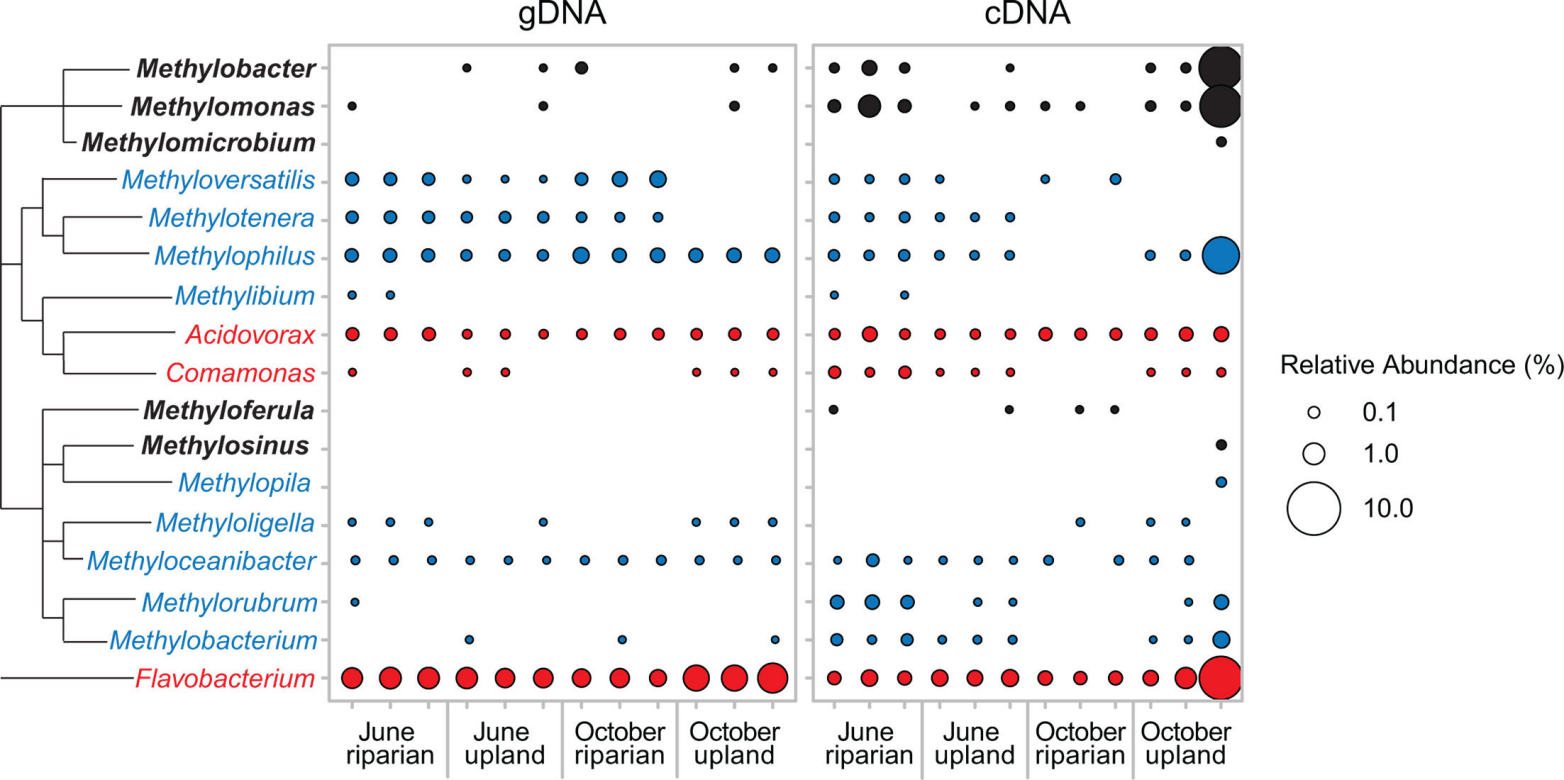
Bubble plot showing the relative abundances (in %) of methanotrophic (bold), NMM (blue), and NMH (red) bacteria calculated relative to the total bacterial community from gDNA and cDNA sequence libraries. Vertical lines on the tree represent shared taxonomic classifications among genera.

We next focused on taxa known to be constituents of methane-oxidizing bacterial communities. We determined the functional classification of ASVs at the genus level, categorizing them as either methanotrophs, NMMs, or NMHs (Table S1). Methanotrophic genera were detected in at least one replicate library of all soil samples, with gDNA libraries containing a lower average relative abundance and diversity of these genera compared to cDNA libraries (Fig. 5). Taxa detected in cDNA, but not gDNA, sequence libraries are known as “phantom taxa”, and their presence may be attributed to various factors, including sample heterogeneity in soil aliquots used for nucleic acid extraction, sampling stochasticity that disproportionately affects relatively rarer taxa such as methanotrophs, or biases in sequencing techniques between DNA and RNA (73, 74). The low methanotrophic abundances in gDNA libraries could be attributed to the low abundance of these bacteria in the soil, which are then represented in cDNA libraries where their relative abundance and potential activity is higher than other microorganisms. Using primers specific for functional genes including the particulate methane monooxygenase gene *pmoA* is a way to profile methane-oxidizing community members more directly (75, 76). The 16S primers used in this study allow for broad, albeit potentially biased, detection of bacteria and archaea (36), enabling us to determine the relative abundance of methane cycling microbes in the overall community and investigate the relevance of non-methylotrophic community members.

Methanotrophic Gammaproteobacteria of the genus *Methylomonas* were detected in nearly all cDNA libraries, whereas the genus *Methylobacter* was specifically found in June riparian and October upland samples. Riparian soil in June had a higher relative abundance of potentially active methanotrophic genera compared to upland soil (average cDNA: 0.58% vs 0.007%). However, in October, upland soil exhibited a greater diversity and higher relative abundance of potentially active methanotrophic genera, including those from Gammaproteobacteria (*Methylobacter*, *Methylomonas*, and *Methylomicrobium*) and Alphaproteobacteria (*Methyloferula* and *Methylosinus*), with *Methylobacter* and *Methylomonas* being particularly abundant. There was no significant correlation (Pearson correlation, *α* = 0.05) between the relative abundance of individual methanotrophs in any library and the median methane flux of the corresponding soil site (Table S2). These findings suggest a complex relationship between the presence or abundance of different methanotrophic genera, with no straightforward correlation indicated by the varied methanotrophic abundances.

**Fig 6:**
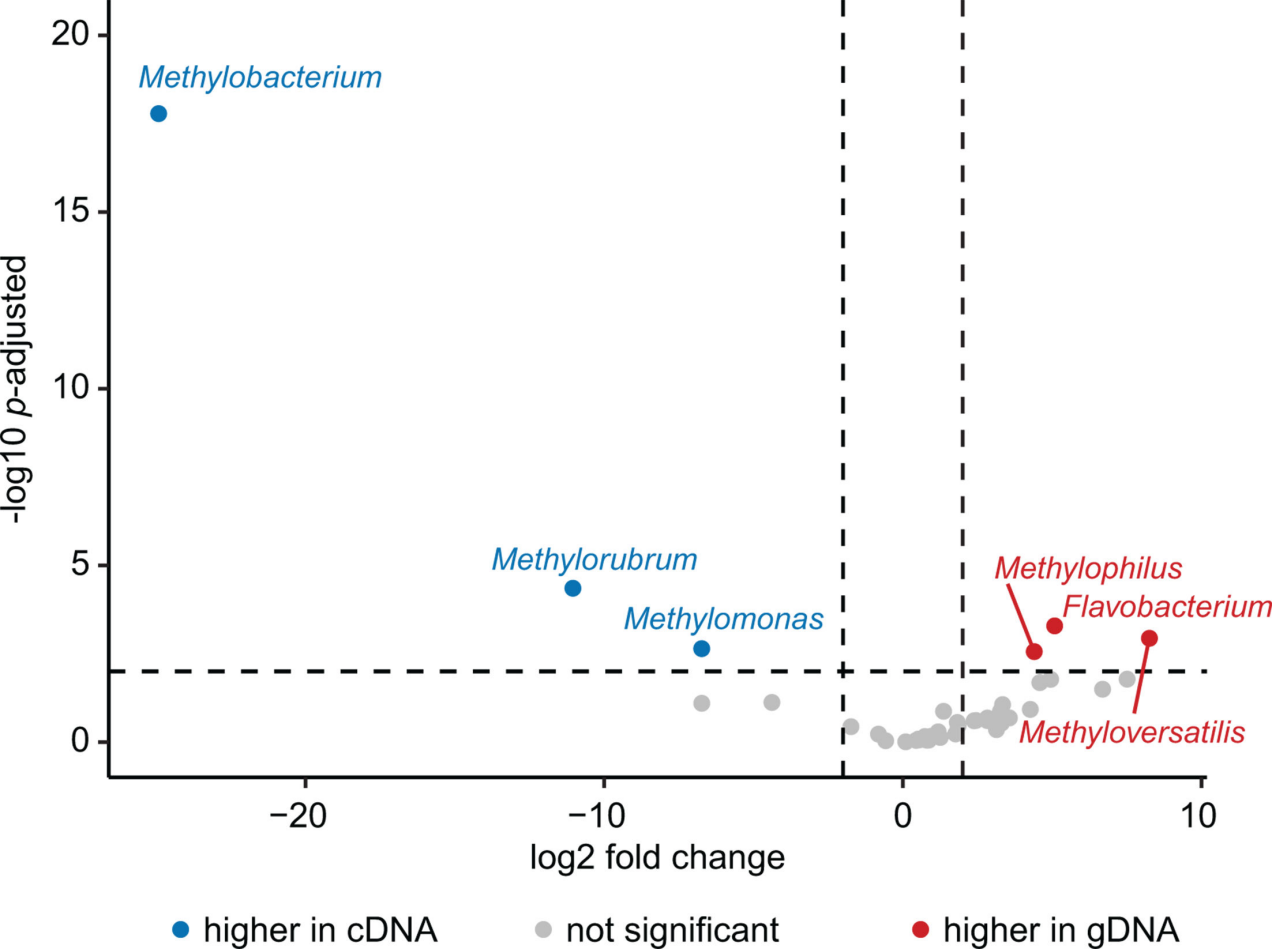
Differential abundance of methane-oxidizing bacterial community members based on ASV counts. Significance determined by |log2(fold change)| < 2 and *P*-adjusted < 0.01 using DESeq2.

To determine the extent to which methane-oxidizing bacterial community members were responsible for the significant differences in community diversity between gDNA and cDNA libraries, we analyzed the relative abundance of taxa in the methane-oxidizing bacterial community across samples. Considering all samples across different months and soil types, the families *Methylococcaceae* (methanotrophs) and *Methylobacteriaceae* (NMMs) were significantly more abundant in cDNA libraries, while *Methylophilaceae* (NMMs), *Flavobacterium*, and *Sterolibacteriaceae* (NMHs) were more abundant in gDNA libraries (|log_2_(fold change)| > 2; *P*-adjusted < 0.05) (Fig. 6). An increased relative abundance in cDNA suggests these organisms are potentially metabolically active, whereas taxa significantly increased in the gDNA libraries are more likely in an inactive or dormant state. Along with other taxa not associated with methane oxidation, these members contributed to the observed differences in gDNA and cDNA library composition, as evidenced more broadly by NMDS ordination (Fig. 4). These trends were similarly reflected in the differential abundance of methane-oxidizing community members between gDNA and cDNA when examining single months, soil types, or individual environmental samples.

**Fig 7:**
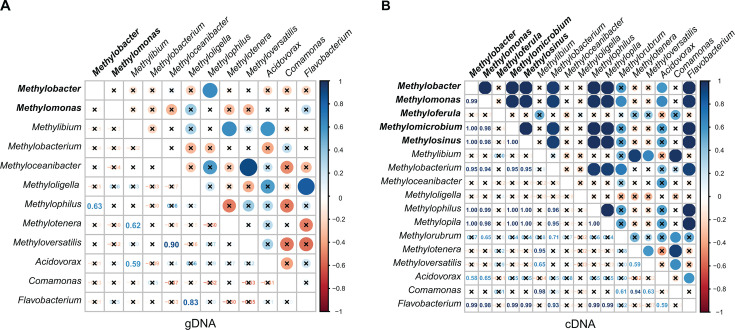
Pearson correlations between methanotrophs (bold), NMMs, and NMHs from genus-level relative abundances derived from (**A**) gDNA and (**B**) cDNA sequence libraries. Pearson correlation coefficients were rounded to two decimal places; insignificant correlations are crossed out (*α* = 0.05). Correlation coefficients and *P* values are listed in Table S3.

Methane-fed microcosms are dominated primarily by methanotrophs from the *Methylococcaceae* family and NMMs from the *Methylophilaceae* family (20, 23, 26). We, therefore, sought to analyze the relationships between methanotrophs, NMMs, and NMHs. In our study, we observed a positive correlation between the relative abundance of the methanotroph *Methylobacter* and the NMM *Methylophilus* in gDNA libraries (*R* = 0.63, *P* < 0.05), indicating a consistent proportional presence of these taxa across different soil types and seasons (Fig. 7A). In the cDNA libraries, we detected significant positive correlations between *Methylococcaceae* family methanotrophs (*Methylobacter* and *Methylomonas*) and various NMMs including those from the *Methylophilaceae* family (Fig. 7B). The relative abundance of *Acidovorax* and *Flavobacterium*, associated NMHs, also positively correlated with *Methylomonas* and *Methylobacter* in the cDNA, but not the gDNA, libraries. We also found positive correlations between the relative abundance of methylotrophs and *Flavobacterium*. The co-occurrence of detectable 16S rRNA gene transcripts in the soil from these genera supports the potential for specific metabolic interactions or crossfeeding within these microbial communities (20–26).

### Conclusion

We investigated the structure and dynamics of methane-oxidizing microbial communities in the sub-alpine forest of Red Butte Canyon, highlighting the relationships that may influence soil methane cycling. Through 16S rRNA gene amplicon sequencing of both gDNA and cDNA from soil samples collected from different soil types at two time points in the growing season, we identified significant variation in the microbial community composition. We found that stable populations (from gDNA libraries) were clearly distinguished between riparian and upland soil, whereas potentially active populations (from cDNA libraries) diverged based on the month. Despite the recognized role of methanotrophic bacteria in mitigating methane flux, these taxa were found in relatively low abundances, underscoring the potential contributions of non-methanotrophic bacteria to methane oxidation processes. This complexity is supported by the observed potential for metabolic partnerships within microbial communities, where positive correlations between methanotrophs from *Methylococcaceae* and NMMs from *Methylophilaceae*, as well as other heterotrophs, point to a network of interactions that may influence methane cycling beyond co-occurrence.

While our findings reveal significant variations among methane-cycling microbial communities across different soils and times, the specific geographic and temporal scope of this study cautions against making broad generalizations. Additionally, though 16S rRNA gene amplicon sequencing effectively identified microbial taxa, it does not encompass all functional aspects of methane cycling. Despite these caveats, our research lays foundational knowledge for understanding soil microbial dynamics and their potential environmental impact. These findings contribute to our understanding of microbe-mediated biogeochemical cycles in forest soils and their potential impact on global methane dynamics, highlighting the need for further *in situ* studies to understand the environmental and ecological influences of methane-oxidizing microbial communities.

## Data Availability

Raw sequence data were deposited in the NCBI Sequence Read Archive (SRA) under the accession number PRJNA1049057.

## SUPPLEMENTAL MATERIAL

Supplemental figures and tables spectrum.00834-24-s0001.pdf Fig. S1 to S5; Tables S1 to S3.

ASM does not own the copyrights to Supplemental Material that may be linked to, or accessed through, an article. The authors have granted ASM a non-exclusive, world-wide license to publish the Supplemental Material files. Please contact the corresponding author directly for reuse.
