# Supplementary material for: Methane-oxidizing bacterial community dynamics in sub-alpine forest soil
Source: Microbiol Spectr. 2024 Sep 17;12(11):e00834-24. doi: 10.1128/spectrum.00834-24 (PMC11537040; doi:10.1128/spectrum.00834-24)
Supplement: Supplemental figures and tables — Fig. S1 to S5; Tables S1 to S3. [file spectrum.00834-24-s0001.pdf]

SUPPLEMENTARY INFORMATION

Methane-oxidizing bacterial community dynamics in sub-alpine forest soil

Delaney G. Beals, Jackson Munn, and Aaron W. Puri*

Department of Chemistry and the Henry Eyring Center for Cell and Genome Science, University of Utah, Salt Lake City, Utah, USA

Keywords: methane flux, methanotrophs, methylotrophs, 16S rRNA, Red Butte Creek

*Corresponding author:
Aaron W. Puri
315 S 1400 E Rm 2020
Salt Lake City, UT 84112
USA
(801) 213-1408
a.puri@utah.edu

TABLE OF CONTENTS

SUPPLEMENTARY FIGURES 3
SUPPLEMENTARY TABLES 8
SUPPLEMENTARY REFERENCES 11

SUPPLEMENTARY FIGURES

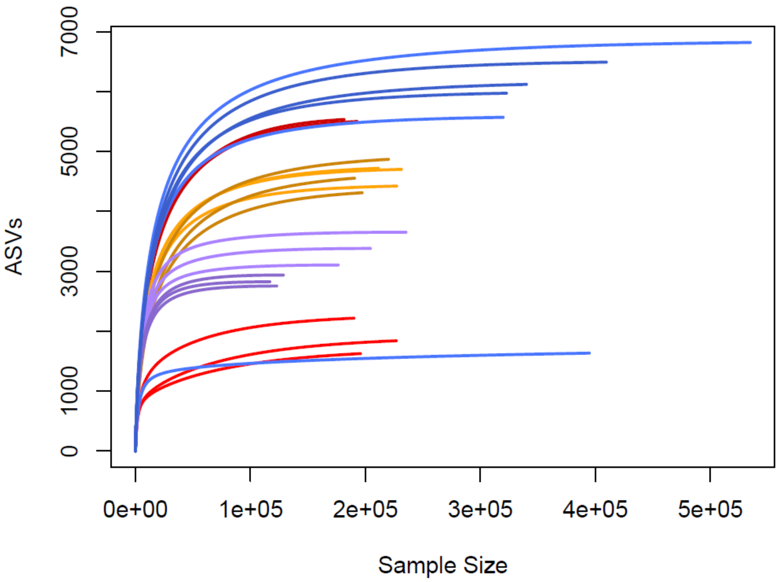

FIGURE S1. Rarefaction plot showing the observed species richness in Red Butte Creek sites: June riparian (red), June upland (orange), October riparian (purple), and October upland (blue). Lighter colors represent cDNA libraries and darker colors show gDNA libraries.

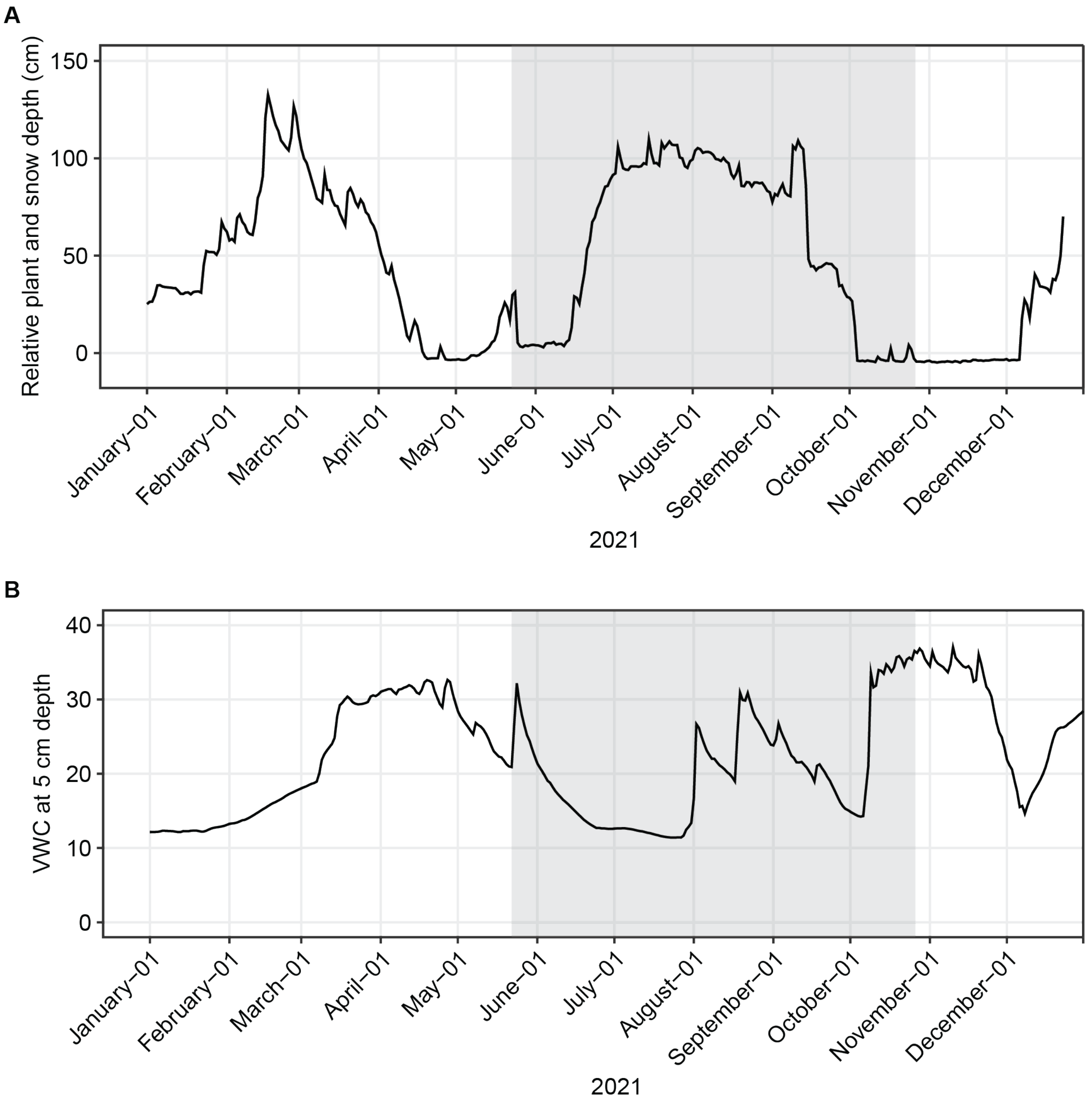

FIGURE S2. Environmental measurements from the Knowlton Fork remote climate research station for the year 2021. Measurements were recorded every 15 minutes. Shaded areas reflect the time period sampled in this study. (**A**) Relative plant height and snow depth were measured using a Judd communications ultrasonic depth sensor, which measures the distance from the sensor to the ground below. Peak plant grow occurred from May to September, while intermittent snowfall and accumulation occurred from January to April and later from October to December. Calculated as the average of six measurements with outliers (>90% of offset or <10) excluded. (**B**) Volumetric water content of upland soil measured using an Acclima Soil Moisture sensor installed 5 cm below the soil surface.

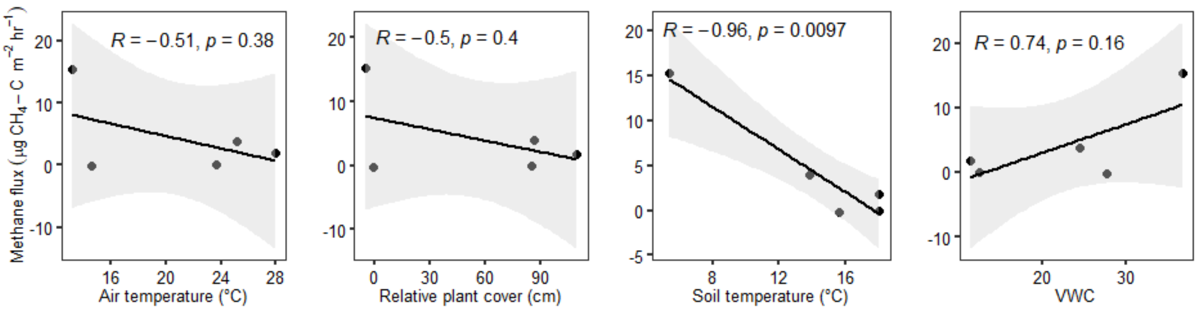

FIGURE S3. Correlation between environmental factors and median methane flux. VWC is volumetric water content. Solid lines are linear regression lines, while the gray shading represents the 95% confidence intervals.

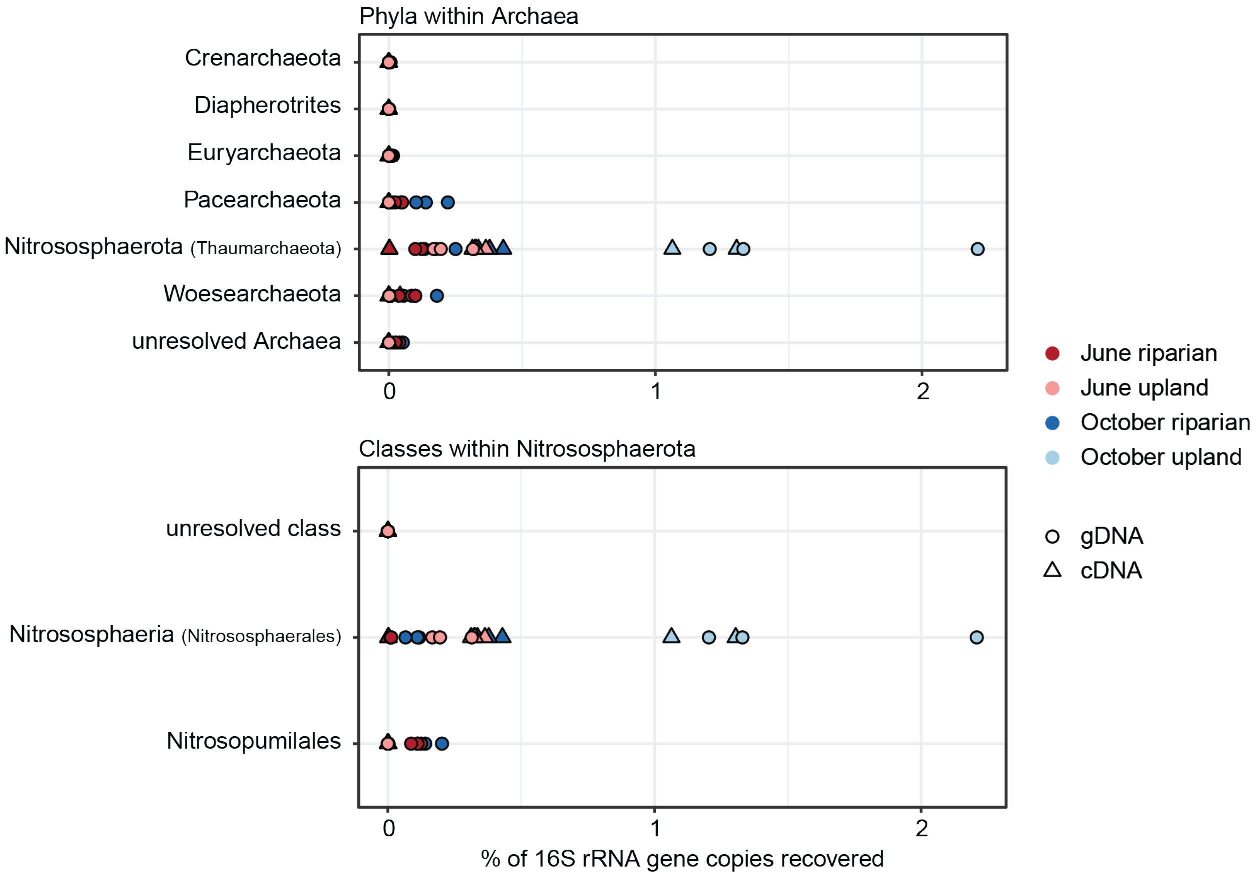

FIGURE S4. Relative abundance of Archaeal phyla and Nitrososphaerota classes from gDNA- and cDNA-derived sequence libraries. Synonyms of taxonomic names are included in parentheses.

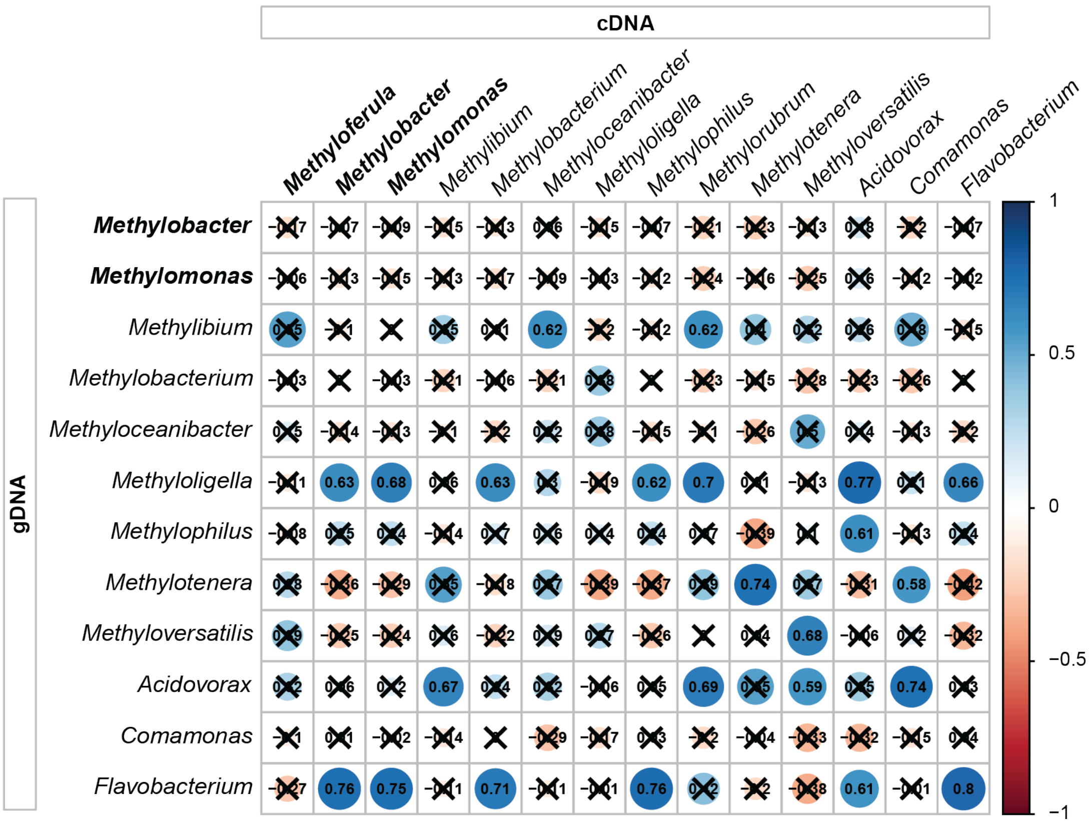

FIGURE S5. Pearson correlations between gDNA- and cDNA-derived relative abundances of methanotrophs (bold), non-methanotrophic methylotrophs, and non-methylotrophic heterotrophs. Pearson correlation coefficients were rounded to two decimal places; insignificant correlations are crossed out (*p* >0.05).

SUPPLEMENTARY TABLES

TABLE S1. Classification of genera identified in this study belonging to the methanotroph, non-methanotrophic methylotroph, and non-methylotrophic heterotroph metabolic groups.

Order	Family	Genus	Reference
		**Methanotrophs**	
Methylococcales	*Methylococcaceae*	*Methylobacter*	(1)
Methylococcales	*Methylococcaceae*	*Methylomonas*	(1)
Methylococcales	*Methylococcaceae*	*Methylomicrobium*	(2)
Hyphomicrobiales	*Beijerinckiaceae*	*Methyloferula*	(3)
Hyphomicrobiales	*Methylocystaceae*	*Methylosinus*	(1)
		**Non-methanotrophic methylotrophs (NMM)**	
Nitrosomonadales	*Sterolibacteriaceae*	*Methyloversatilis*	(4)
Nitrosomonadales	*Methylophilaceae*	*Methylotenera*	(5)
Nitrosomonadales	*Methylophilaceae*	*Methylophilus*	(6)
Burkholderiales	*Comamonadaceae*	*Methylibium*	(5)
Hyphomicrobiales	*Methylocystaceae*	*Methylopila*	(7)
Hyphomicrobiales	*Hyphomicrobiaceae*	*Methyloligella*	(7)
Hyphomicrobiales	*Hyphomicrobiaceae*	*Methyloceanibacter*	(7)
Hyphomicrobiales	*Methylobacteriaceae*	*Methylorubrum*	(8)
Hyphomicrobiales	*Methylobacteriaceae*	*Methylobacterium*	(9)
		**Non-methylotrophic heterotrophs (NMH)**	
Burkholderiales	*Comamonadaceae*	*Acidovorax*	(10)
Burkholderiales	*Comamonadaceae*	*Comamonas*	(11)
Flavobacteriales	*Flavobacteriaceae*	*Flavobacterium*	(12)

TABLE S2. Pearson correlation analysis of methanotroph relative abundance in relation to soil temperature, methane flux, and gravimetric water content (GWC). Soil temperature and methane flux were measured at the time and site of soil collection and GWC for each soil was determined in the lab.

	**gDNA**					
	CH_4 flux		Soil temperature		GWC	
	R	*P* value	*R*	*P* value	*R*	*P* value
Methylobacter	0.505	9.42E-02	-0.307	3.32E-01	-0.166	6.06E-01
Methylomonas	-0.202	5.28E-01	0.351	2.63E-01	-0.280	3.78E-01
	**cDNA**					
	CH_4 flux		Soil temperature		GWC	
	R	*P* value	*R*	*P* value	*R*	*P* value
Methylobacter	-0.135	6.75E-01	0.255	4.25E-01	-0.282	3.74E-01
Methylomonas	-0.170	5.97E-01	0.211	5.11E-01	-0.196	5.42E-01
Methylosinus	-0.122	7.06E-01	0.265	4.05E-01	-0.306	3.33E-01
Methylomicrobium	-0.122	7.06E-01	0.265	4.05E-01	-0.306	3.33E-01
Methyloferula	0.054	8.69E-01	-0.375	2.30E-01	0.404	1.93E-01

TABLE S3. Pearson correlation analysis between the relative abundance of methanotrophic (columns) and non-methanotrophic (rows) genera. Shaded cells reflect *p* <0.05.

	gDNA			
	Methylobacter		*Methylomonas*	
	R	*P* value	*R*	*P* value
Methylibium	-0.1549	6.31E-01	-0.1239	7.01E-01
Methylobacterium	-0.1441	6.55E-01	-0.2029	5.27E-01
Methyloceanibacter	0.0994	7.59E-01	-0.3350	2.87E-01
Methyloligella	-0.2474	4.38E-01	0.3587	2.52E-01
Methylophilus	0.6321	2.75E-02	0.0379	9.07E-01
Methylotenera	-0.0933	7.73E-01	-0.3041	3.36E-01
Methyloversatilis	0.0424	8.96E-01	-0.3241	3.04E-01
Acidovorax	-0.2143	5.04E-01	0.0848	7.93E-01
Comamonas	-0.2311	4.70E-01	-0.0903	7.80E-01
Flavobacterium	-0.2141	5.04E-01	0.2522	4.29E-01

	cDNA					
	Methylobacter		*Methylomonas*		*Methyloferula*	
	R	*P* value	*R*	*P* value	*R*	*P* value
Methylibium	-0.1337	6.79E-01	-0.1271	6.94E-01	0.5004	9.75E-02
Methylobacterium	0.9505	2.15E-06	0.9422	4.62E-06	0.0127	9.69E-01
Methyloceanibacter	-0.1165	7.18E-01	0.0103	9.75E-01	-0.2187	4.95E-01
Methyloligella	-0.1447	6.54E-01	-0.1675	6.03E-01	0.1438	6.56E-01
Methylophilus	0.9991	3.74E-15	0.9851	5.66E-09	-0.1498	6.42E-01
Methylorubrum	0.5678	5.42E-02	0.6468	2.30E-02	0.1829	5.69E-01
Methylotenera	-0.2051	5.22E-01	-0.1808	5.74E-01	0.4170	1.77E-01
Methyloversatilis	-0.2122	5.08E-01	-0.1924	5.49E-01	0.3885	2.12E-01
Acidovorax	0.5806	4.78E-02	0.6470	2.30E-02	-0.3081	3.30E-01
Comamonas	-0.0467	8.85E-01	-0.0212	9.48E-01	0.5124	8.85E-02
Flavobacterium	0.9925	1.90E-10	0.9776	4.25E-08	-0.1995	5.34E-01

SUPPLEMENTARY REFERENCES

1. Whittenbury R, Phillips KC, Wilkinson JF. 1970. Enrichment, isolation and some properties of Methane-utilizing Bacteria. *J Gen Microbiol* 61:205–218.
2. Kalyuzhnaya M, Khmelenina V, Eshinimaev B, Suzina N, Nikitin D, Solonin A, Lin J-L, McDonald I, Murrell C, Trotsenko Y. 2001. Taxonomic characterization of new alkaliphilic and alkalitolerant methanotrophs from soda lakes of the southeastern Transbaikal region and description of *Methylomicrobium buryatense* sp. nov. *Syst Appl Microbiol* 24:166–176.
3. Dedysh SN, Naumoff DG, Vorobev AV, Kyrpides N, Woyke T, Shapiro N, Crombie AT, Murrell JC, Kalyuzhnaya MG, Smirnova AV, Dunfield PF. 2015. Draft genome sequence of *Methyloferula stellata* AR4, an obligate methanotroph possessing only a soluble methane monooxygenase. *Genome Announc* 3:e01555-14.
4. Kalyuzhnaya MG, De Marco P, Bowerman S, Pacheco CC, Lara JC, Lidstrom ME, Chistoserdova L. 2006. *Methyloversatilis universalis* gen. nov., sp. nov., a novel taxon within the Betaproteobacteria represented by three methylotrophic isolates. *Int J Syst Evol Microbiol* 56:2517–2522.
5. Chistoserdova L. 2011. Modularity of methylotrophy, revisited. *Environ Microbiol* 1 3:2603–2622.
6. Jenkins O, Byrom D, Jones D. 1987. *Methylophilus*: A new genus of methanol-utilizing bacteria. *Int J Syst Evol Microbiol* 37:446–448.
7. Martineau C, Mauffrey F, Villemur R. 2015. Comparative analysis of denitrifying activities of *Hyphomicrobium nitrativorans*, *Hyphomicrobium denitrificans*, and *Hyphomicrobium zavarzinii*. *Appl Environ Microbiol* 81:5003–5014.
8. Green PN, Ardley JK. 2018. Review of the genus *Methylobacterium* and closely related organisms: a proposal that some Methylobacterium species be reclassified into a new genus, *Methylorubrum* gen. nov. *Int J Syst Evol Microbiol* 68:2727–2748.
9. Patt TE, Cole GC, Hanson RS. 1976. *Methylobacterium*, a new genus of facultatively methylotrophic bacteria. *Int J Syst Evol Microbiol* 26:226–229.
10. Beck DAC, Kalyuzhnaya MG, Malfatti S, Tringe SG, Glavina del Rio T, Ivanova N, Lidstrom ME, Chistoserdova L. 2013. A metagenomic insight into freshwater methane-utilizing communities and evidence for cooperation between the *Methylococcaceae* and the *Methylophilaceae*. *PeerJ* 1:e23.
11. Yu Z, Groom J, Zheng Y, Chistoserdova L, Huang J. 2019. Synthetic methane-consuming communities from a natural lake sediment. *mBio* 10:e01072-19.
12. Zheng Y, Wang H, Yu Z, Haroon F, Hernández ME, Chistoserdova L. 2020. Metagenomic insight into environmentally challenged methane-fed microbial communities. *Microorganisms* 8:1614.
